# Homeopathy, the Law of Cure and the Basis of a True Psychology

**Published:** 1897-07

**Authors:** J. W. Thomson

**Affiliations:** 159 W. 48th St., New York City


### HOMEOPATHY, THE LAW OF CURE AND THE BASIS OF A TRUE PSYCHOLOGY.

J. W. Thomson, M. D., Author of "The Philosophy of Homeopathy."

For many reasons people see things differently. Truth is many-sided. Like the two sides of the shield unlike views may be partial rather than inaccurate. Still there must be a broad line of demarkation between truth and error. It is to be regretted that there should be ideas advanced in your article on "The Artistic Spirit in Medicine" * that seems to me to belong to the latter category, and thus not in line with pure Homeopathy. You say on page 11, "There is something admirable even in disease." I have never been able to discover it. My thought has always been how best to get rid of the disorderly incubus. If you were called to a patient and

* See "The Artistic Spirit in Medicine," by Stewart Close, M. D., in The Homeopathic Physician for January, page 9.

said you found something so "admirable" in certain phases of the disease that you would recommend the patient to retain at least a part of the disease, and you would endeavor to cure the remainder, this would be logical from your standpoint. For surely we should not try to eradicate anything we esteem "admirable."

You continue, "a symptom picture of disease * * * is a thing of orderly growth and development." I maintain, on the contrary, that disease is always, in a greater or less degree, the destruction of order. Anything "orderly" or "admirable" is not in the disease, *per se,* but is the struggles of the vital force against the destructive, the diabolical negations of disease.

Disease is not, as you affirm, "a harmonious whole, giving intelligible expression to an idea." It is inharmonious, it is the distunement, it is the breaking up of the "harmonious whole" that constitutes the play, so to speak, of the life force in its tenement of flesh. Imagine syphilis, small-pox, or leprosy, "giving intelligible expression to an idea."

What you term "the growth of a picture" is not akin to the development of disease. A picture by a true artist is the thought, the expression, the interpretation of some phase or phases of nature or humanity, or perhaps the limning of some other arts and portrayed on canvas in harmony with the genius and artistic ability of the artist. The growth of the picture may be said to be "orderly," the result "admirable." There are, fortunately, many of these *chefs d'oeuvre* sacredly preserved, and whose destruction would be looked upon as calamitous. On the contrary, "a symptom picture" is something that the true physician strives to exterminate as speedily as may be, and that the patient earnestly desires to be freed from—if he be in his right mind—or if pain and suffering have not made him desirous to shuffle off this mortal coil, and reach that bourne "whence no traveler returns."

I much question if there be such a manifestation as a "natural symptom picture." Disease—as I have intimated—is disorderly, it is the negation of health. Its tendency is to destroy all that is most to be desired. The true natural state and condition is health; disease being abnormal is, therefore, unnatural. When health is restored we have the natural condition. There cannot be "consistency, coherency, and character" in disease, because disease rends, deprives of beauty, strength, symmetry. We might as well speak of "consistency, coherency, and character" in a lie, which is the negation of the truth, as disease is the negation of health. Evil and false, whether in the physical or moral world, would bring chaos, and must be kept in subjection by the good and true, otherwise they would o'errun all bounds and cause universal desolation.

On page 15 you say, "unprejudiced study of the phenomena of hypnotism, telepathy, spiritism, mental suggestion, 'mind cure,' 'Christian science,' 'faith cure,' hysteria, and psychic phenomena in general by men of high attainments and true scientific spirit, has done more to advance the science of psychology in the last ten years than in a century before." Then you continue, "all this is fertile and productive ground for the Hahnemannian, for it familiarizes him with the phenomena and laws of the human mind, and enables him to make a better examination of his patients." How all this can help the Hahnemannian I fail to see. If all your laudation of these things were true, which it is not, they would be of no more use to Homeopathy than to mathematics or chemistry. Homeopathy, like all other sciences, stands on the broad foundation of its own principles, philosophy, and practice. Its facts were first discovered. I open Bohn's edition of *Bacon's Physical and Metaphysical Works*, 1886, and from page 184 extract the following: "Celsus, a wise man, as well as a physician, gravely and ingenuously acknowledges that medicine and cures were first discovered, and the reasons and causes of them discovered afterwards, not that causes, first derived from the nature of things, gave light to the invention of cures and remedies." It is built upon facts. Homeopathic facts and none others. The facts of the provings must agree with the facts of the symptoms. In all other so-called systems of medicine there is doubtless room for imagination, in Homeopathy it is the facts, and the facts alone, that are indispensably necessary.

I do not believe that one human being ought voluntarily, or be made involuntarily, to subject his mind to the will and consciousness of another, either by hypnotism or any other way. In the case of painful operations the use of anesthesia does not subject the patient's mind to the will and mind of another. It is temporary unconsciousness. Yet sometimes the patient is subject to hallucination; therefore, and for other reasons, there ought always to be at least one witness in addition to the operator. Albert Noll, of Berlin, an authority on hypnotism, says: "The subject is completely without a will of his own." Has any one the right to assume such responsibility? I trow not. There are moral questions superior even to the attainment and conservation of health. I read in a New York paper that a hypnotist, Prof. Ferris, at Hamilton, Ont., on May 12th last, put Fred Smith under the influence, placed him in a coffin, and buried him in a vacant lot, with a ventilating shaft leading to the grave. Poor Smith was to have been kept under the diabolism—I beg pardon, under the hypnotic charm—for three days. After the burial, people surrounded the grave. The poor victim could be heard crying: "For God's sake let me out of here." Willing hands quickly unearthed him. The Professor explained that Smith had not been sufficiently hypnotized. It would be too horrible a punishment to put Prof. Ferris in the same grave for three days, of course sufficiently hypnotized, and I do not believe in the Delaware law of corporal castigation, yet I would be willing for this time only to make an exception in favor of Prof. Ferris, upon condition that the cat-o-nine-tails was administered so severely to his bare back that he would remember it for the term of his natural life. Now do not mistake me about Prof. Ferris. My thought is purely a judicial one. If he could be persuaded that his course of conduct was all wrong and give up the hypnotic craze for wholesome studies, the true purpose would be gained. The truth is before everything. With many, if their personal ends and preferences are served, that seems to be all they care for. All I contend is that this moral turpitude, this cold-blooded scoundrelism in the name of science, ought to be put an end to. They are experimenting with hypnotism on patients in some of the allopathic hospitals, some are also striving to make their patients' ailments agree with their theories on antitoxin. Procrustean like, they endeavor to make therapeutics adapt itself to their peculiar views. All this is in line with their ideas of the "true scientific spirit," but it has not anything in common with the Homeopathy of Hahnemann.

Now, with reference to "Christian science." Mrs. Mary Baker E. Eddy, in preface to *Science and Health*, page xi, says: "Many imagine that the phenomena of physical healing in Christian science presents a phase of the action of the human mind, which, in some unexplained way, results in the cure of sickness. On the contrary, Christian science rationally explains that all other pathological methods are the fruits of human faith in matter, in the workings not of spirit, but of the fleshly mind, which must yield to science." It is to be observed that "Christian Science," according to Mrs. E. is "physical healing," that it is a "pathological method," is the "workings" of the "fleshly mind." Is it not a revelation that the "mind" is "fleshly"? "Fleshly mind." Is not this crass materialism in the name of Christianity and science? It might be suggested that therapeutical and not pathological would be the correct term in this connection. But that may "be a delusion of the 'fleshly mind,' which must yield to science."

How opposed to the teachings of Hahnemann this "physical healing" and "fleshly mind" business is may be shown by a quotation from *The Organon*. In note to paragraph 31 Hahnemann says: Diseases "are not mechanical or chemical changes of the material substance of the body, that they do not depend upon a morbific material principle, and that they are solely spiritual and dynamic changes of the animal economy."*

Preface, page xii, is the following: "Note—the author takes no patients, and declines medical consultation." May we not say in the language of Shakespeare:

"O, most lame and impotent conclusion."

It was not thus that Hahnemann promulgated the law of cure he discovered. It was by relieving and curing the sick. He fought with the fire of love and the sword of truth, and he left the knowledge of the law a heritage to the human race.

On page 39 is written: "The schools have rendered faith in drugs the fashion rather than faith in Deity." In common with many other benighted mortals I have had the thought that faith, born of the knowledge of the uses that the Deity has implanted in drugs does not mitigate against faith in Him. But it appears to be only a matter of "fashion."

Then on page 42, "Physiology exalts matter and dethrones mind, and pretends to rule man by material law instead of spiritual," and again, "Medicine is not a science, but a bundle of speculative human theories." There is no explanation of how mind is dethroned and matter exalted by physiology. Law is not inherent in matter. All laws act upon matter. Thus it is the life force that acts upon the body, keeping it

* Microbiology, the modern craze, puts effect for cause; to the low forms of life that are the result of disease and death of tissue are assigned the rank of producers of abnormal conditions, which is equivalent to saying that the blow-flies on the dead horse were the cause of his demise.

in health by the orderly performance of all its functions. These functions we call physiology. When the life force leaves the body it becomes subject to chemical law. Physiology, then, is the action of the life force on the body as a whole, and upon all the component parts thereof. It is the universal enthronement of the mind throughout the body.

That Homeopathy is included in the medicine which is not science is shown on page 46: "But the drug is attenuated to such a degree that not a vestige of it remains. Thus we learn that it is not the drug which expels the disease, or changes one of its symptoms." Thus, according to *Science and Health*, homeopathic practitioners are either deceivers or themselves deceived, knaves or fools. And this is one of the things that you think "has done more to advance the science of psychology in the last ten years than in a century before." The author of *Science and Health* takes a diametrically opposite view of the value of spiritism that you entertain. Thus, page 237, she says it has "no scientific basis or origin in principle, and having no proof or power outside of human testimony or belief, etc." Is it not a legitimate inference that Christian science has something to offer "outside of human testimony and belief," and also that modern spiritualism has, not as you suppose, added anything to our knowledge of psychology? There are places in this book in which the author seems not only to have drawn largely upon her imagination, but even to have lapsed into incoherency.

Reverting again, for a moment, to hypnotism, what true knowledge could ever be gained by "gazing at the tip of the nose" until you are insensible. How any rational being could be led away by such idiotic drivel seems beyond comprehension. You will find more true psychology in *The Genius of the Homeopathic Healing Art*, by one Dr. Samuel Hahnemann, published in 1833, than in all that has been brought to light since.

Again Homeopathy is the true "mind cure," all others are sporadic or ephemeral. Homeopathy answers affirmatively the question propounded by Avon's bard:

"Canst thou minister to a mind diseased?"

Of course we are not now speaking of what may be accomplished by a pure, healthy mind, and well-regulated life in warding or throwing off disease, yet, even in such case, Homeopathy will be of use because it is God's law of cure.

159 W. 48th St., New York City.

